# Influence of meteorological conditions on herpes zoster occurrence: a retrospective cohort study

**DOI:** 10.3389/fmed.2025.1643828

**Published:** 2025-10-10

**Authors:** Michał Ochal, Katarzyna Glińska Lewczuk, Ewa Dragańska, Iwona Cymes, Jerzy Romaszko

**Affiliations:** ^1^Department of Family Medicine and Infectious Diseases, Collegium Medicum, School of Medicine, University of Warmia and Mazury in Olsztyn, Olsztyn, Poland; ^2^Department of Water Management and Climatology, Faculty of Agriculture and Forestry, University of Warmia and Mazury in Olsztyn, Olsztyn, Poland

**Keywords:** herpes zoster, climate, Universal Thermal Climate Index (UTCI), temperature, weather, epidemiology

## Abstract

**Introduction:**

Herpes zoster is caused by the reactivation of the varicella zoster virus, typically affecting older adults and immunocompromised individuals. Although various studies have examined potential triggers, the influence of meteorological conditions on herpes zoster reporting remains unclear. We aimed to investigate whether meteorological and biometeorological factors, including the Universal Thermal Climate Index (UTCI) and the H index (air cooling power), have an effect on herpes zoster occurrence.

**Methodology:**

We performed a retrospective analysis of medical records for herpes zoster cases (ICD-10 code B02) from 2009 to 2023 at two medical facilities in northeastern Poland. Local meteorological data were obtained from regional stations. Statistical analyses accounted for patient age, seasonality, and thermal conditions (as measured by UTCI).

**Results and discussion:**

No seasonal pattern in herpes zoster reporting was observed, and there were no statistically significant correlations between basic meteorological variables (ambient temperature, relative humidity, wind speed, etc.) and the number of cases. However, results showed that under strong heat stress conditions (UTCI class 8), the risk of herpes zoster was approximately 20–25% higher compared to thermally neutral days.

**Conclusion:**

Herpes zoster occurrence does not exhibit a seasonal pattern. Composite indices like the UTCI and the H index can be useful tools for predicting the risk of herpes zoster. The relative risk of herpes zoster is lower during cold stress conditions and higher during heat stress conditions.

## Introduction

Herpes zoster (shingles) is caused by reactivation of the varicella zoster virus (VZV), which remains latent in sensory ganglia after a primary varicella (chickenpox) infection (1). Primary VZV infection can be especially severe in adults and immunocompromised individuals (2), but after recovery the virus adopts a dormant state instead of being eliminated from the body (3). Virus reactivation into herpes zoster is often precipitated by a weakened immune system due to factors such as aging, stress, chronic disease, or immunosuppressive therapy (4–6). In particular, cell-mediated immunity wanes with age, reducing the body’s ability to contain the virus and thereby increasing the risk of reactivation. A crucial role in maintaining VZV latency is played by CD8 + T lymphocytes. These immune cells provide surveillance by secreting cytokines such as interferon-gamma (IFN-*γ*), which enhance neuronal resistance to viral replication (7). Additionally, pro-inflammatory cytokines (interleukin-1, interleukin-6, and tumor necrosis factor-*α*) are implicated in VZV reactivation: elevated levels of these mediators in response to stress or infection can disrupt the neuronal environment, promoting viral replication (8). Clinically, herpes zoster manifests as a painful vesicular rash with fluid-filled blisters that eventually rupture, crust, and heal. The rash is usually limited to one side of the body, reflecting the reactivation of the virus in a specific sensory ganglion (dermatomal distribution) (9). The virus in the lesions is transmissible; contact with the vesicles can cause varicella in susceptible individuals. A common complication of herpes zoster is postherpetic neuralgia (PHN), a chronic neuropathic pain that can persist for months or even years after the rash resolves (10). The risk of PHN is particularly high in older patients (10).

Considerably less is known about the influence of environmental factors especially weather conditions—on the reactivation of VZV (11). For example, one study found that vitamin D supplementation in hemodialysis patients was associated with a lower occurrence of herpes zoster, suggesting that vitamin D deficiency (potentially linked to reduced sun exposure in winter) might increase reactivation risk (12). In contrast, an analysis by Berlinberg et al. (USA) noted a peak in herpes zoster cases during the summer months (13), implying a possible seasonal effect. Other studies have explored specific meteorological factors: Choi et al. (South Korea) reported that higher ambient temperatures were associated with increased zoster risk, consistent with a heat-stress effect on immunity (14), whereas Yang et al. (China, Shanghai) observed more frequent herpes zoster cases in winter, when colder temperatures and reduced sunlight (hence lower vitamin D levels) might weaken immune defenses (15). Notably, Kawai et al. (USA) found that higher ambient ultraviolet radiation (UVR) exposure was associated with an increased risk of herpes zoster in men, suggesting that UVR-induced immunosuppression may play a role in VZV reactivation (16). It should be noted that these studies quantified thermal stress using single meteorological parameters, such as mean air temperature or UV radiation, rather than composite indices. Such methodological differences may contribute to the heterogeneity of reported findings and highlight the advantage of using the UTCI in our analysis. These inconsistencies across studies highlight that the impact of meteorological factors on herpes zoster remains unclear and potentially context-dependent. Differences in study populations, geographic regions, climate types, and methodologies may explain why some studies found seasonal or thermal effects while others did not. This unresolved scientific picture underscores the need for further research, particularly in regions with moderate continental climates, where such analyses remain limited. Understanding whether and how weather conditions affect herpes zoster reporting is important for public health, as this knowledge could inform new prevention strategies for high-risk groups (17). Moreover, with global climate change leading to more frequent extreme weather events, clarifying the impact of meteorological factors on health is becoming increasingly crucial (18). In Poland, recent decades have witnessed a growing occurrence of extreme weather events, reflecting broader climate variability in Central Europe (19). Although the pan-European heatwave of 2003 had relatively limited direct mortality impact in Poland, the record warm summers of 2010 and several other heatwaves (1992, 1994, 2006, 2014, 2015) did significantly affect public health (19). Poland has also experienced devastating flood events, including the “Millennium Flood” of 1997 and the widespread inundations in 2010, both associated with intense and prolonged precipitation (20). Additionally, meteorological droughts have been documented in Polish river catchments between 1981 and 2010, impacting water resources and agricultural productivity (21). These events illustrate how, despite a temperate continental climate, Poland already contends with climate extremes that are relevant to interpreting meteorological influences on health outcomes.

In the context of herpes zoster, shifting climate patterns may alter any seasonal occurrence trends and could even exacerbate disease occurrence under specific conditions (22, 23). In light of these considerations, the aim of this study was to analyze the effect of meteorological and biometeorological conditions on the occurrence of herpes zoster. The concept of ‘biometeorological’ conditions refers to the interdisciplinary field of biometeorology, which investigates how weather and climate interact with human physiology and health, and provides the framework for applying indices such as the UTCI (24) or air cooling power (H) (25). This work specifically evaluates whether composite weather indices (such as the UTCI) are associated with changes in herpes zoster occurrence, thereby providing a foundation for future research toward more effective preventive strategies.

## Materials and methods

### Study population

In this retrospective study, data were collected from two medical facilities in northeastern Poland: the Non-Public Health Care Centre “Pantamed” in Olsztyn and the Władysław Biegański County Hospital in Iława (including its Night and Holiday Emergency Health Care Unit, NiŚOZ). In Poland, Night and Holiday Emergency Health Care Units (NiŚOZ) provide urgent outpatient care during nights, weekends, and public holidays, functioning similarly to urgent care centers in other healthcare systems (26). The two sites are about 70 km apart. Pantamed serves approximately 13,000 registered patients (providing ~60,000 consultations per year), while the Iława hospital’s NiŚOZ unit serves a county population of about 90,000, with 15,595 patient admissions to NiŚOZ recorded during the study period (27). From these facilities’ records, we extracted all diagnosed cases of herpes zoster (ICD-10 code B02) in adults for the years 2009–2023 (28). Information on comorbidities and other diagnoses was available in medical records but was not systematically included as covariates in the present analysis. For each case, the date of diagnosis, patient age, sex, and relevant medical history (e.g., prior visits and diagnoses) were recorded. We excluded repeat entries for the same patient’s zoster episode within a 30-day period to avoid double-counting. In total, 1,102 unique adult cases of herpes zoster were analyzed.

### Climate characteristics

Meteorological data for the period 2009–2023 were obtained from the Institute of Meteorology and Water Management station in Olsztyn (Poland). According to the standards of the World Meteorological Organization (WMO, 2017) and the Polish Institute of Meteorology and Water Management (IMGW-PIB, 2015), synoptic stations provide measurements representative of the broader geographical area; therefore, the Olsztyn station data were considered reliable for both Olsztyn and Iława (29, 30). According to the updated Köppen–Geiger climate classification, this region has a cold continental climate without a dry season and with a warm summer (Dfb) (31). The daily meteorological parameters collected included air temperature (daily mean, maximum, and minimum), wind speed, relative humidity, and atmospheric pressure. We used these data (specifically the measurements at 12:00 UTC each day for temperature, humidity, vapor pressure, cloud cover, and wind) to calculate the UTCI for each day. It is expressed in °C and represents a composite indicator derived from a human heat balance model that integrates meteorological variables to quantify cold and heat stress (32). UTCI has previously been linked to adverse health effects, including increased mortality, cardiovascular hospitalizations, and, in emerging evidence, infectious diseases (33). In particular, Di Napoli et al. (Europe-wide study) demonstrated across Europe that UTCI values above moderate to strong stress levels (approximately 26–32 °C) were associated with elevated death counts, underscoring its relevance in capturing heat-related health risks (33).

The UTCI is expressed in °C and reflects the integrated effect of weather conditions on the human body’s thermal balance (34). Based on UTCI values, thermal stress levels are categorized into classes. Cold stress conditions occur at low temperatures combined with wind (UTCI classes 1–5, corresponding to ≤9.0 °C), while heat stress occurs at high temperatures with intense solar radiation (heat stress classes 7–10, >26.0 °C). Conditions in the intermediate range (UTCI 9.1–26.0 °C, class 6) indicate thermal comfort (no significant heat or cold stress) (24, 35). In our dataset, the extreme UTCI classes 1, 9, and 10 did not occur during the study period and were thus excluded from analysis. In addition to UTCI, we calculated the H index (air cooling power, expressed in W/m^2^) for each day at 12:00. This index quantifies the rate of heat loss from a unit surface of the human body due to convection and conduction under the influence of air temperature and wind speed (31). We applied two classes of the cooling power index: cold (H > 1,260 W/m^2^) and very hot (H < 210 W/m^2^). Both UTCI and H index values were computed using the BioKlima software (36).

### Statistical analysis

Analyses of distribution and group differences were performed using Statistica v13.0 (Dell Inc., Tulsa, USA). Data distribution within groups was first assessed with the Shapiro–Wilk test for normality (*p* < 0.05 indicating significance). Homogeneity of variances was evaluated by Levene’s test (*p* < 0.05). Since most variables did not meet the assumption of normal distribution and homoscedasticity, we employed non-parametric methods. To compare differences among groups (e.g., monthly case numbers, age groups, or classes based on UTCI and H) we applied the Kruskal–Wallis one-way analysis of variance. When significant overall differences were found, Dunn’s *post hoc* test with Bonferroni correction was applied for multiple pairwise comparisons. A two-tailed *p*-value <0.05 was considered statistically significant for all tests. In addition, Spearman’s rank correlation coefficients were computed to assess the relationships between the number of herpes zoster cases and individual meteorological variables (e.g., temperature, humidity, precipitation, wind) as well as the UTCI and H. This correlation analysis was used to gage the strength and direction of any linear associations between weather factors and zoster occurrence.

To examine how the occurrence of herpes zoster relates to multiple meteorological variables simultaneously, we performed a multivariate analysis using Principal Coordinate Analysis (PCoA) in XLSTAT by Lumivero (version 2023.2.0) (37). PCoA was appliedto visualize similarities or dissimilarities in the dataset. Prior to the analysis, variables measured on different scales were standardized and square-root transformed to reduce skewness. We constructed a Euclidean distance matrix, and PCoA was then carried out to identify principal axes of variation. PCoA provided the percentage of variance explained by each axis, allowing us to evaluate how well the reduced-dimensional representation captured the structure of the original multivariate data.

Furthermore, we calculated the relative risk (RR) of herpes zoster under different thermal conditions defined by UTCI. Specifically, we compared the risk of herpes zoster during periods of cold stress and heat stress with the risk under thermoneutral conditions [UTCI class 6 (9–26 °C)] which served as the reference category for the total cohort and separately for patients aged >65 years. In addition to RR values with corresponding 95% CIs, we also report Z-scores and *p*-values obtained from pairwise comparisons. We considered an RR statistically significant if the 95% confidence interval (CI) did not include 1. RR calculations were performed using PAST software (version 4.03). We chose to use RR (a risk ratio) in this context, rather than an odds ratio, because our data encompassed the entire population at risk in the study period rather than a case–control sample. Using RR provides a more direct measure of the effect size of thermal stress on disease occurrence in this retrospective cohort analysis.

## Results

**Figure 1:**
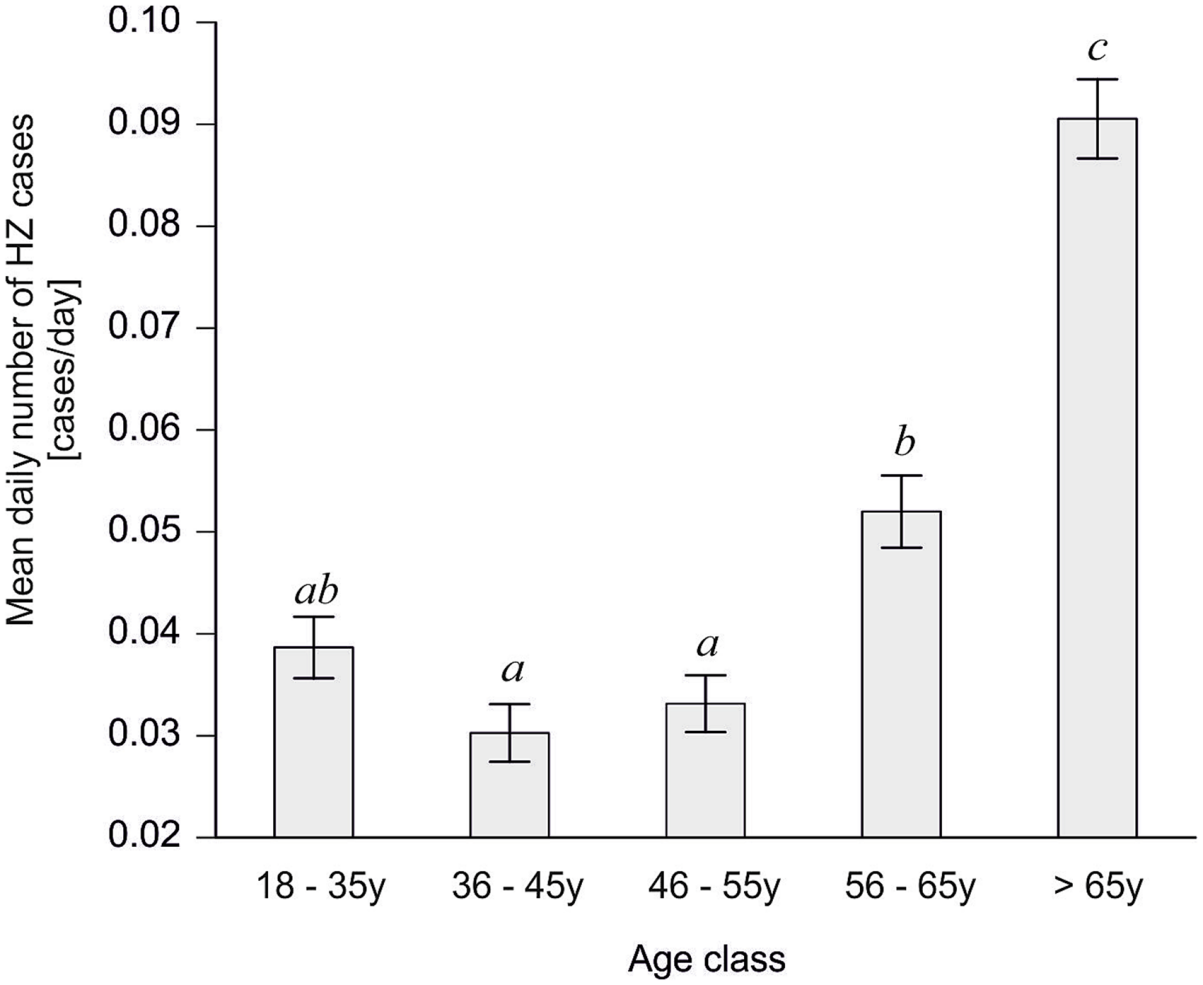
Average number of admissions for herpes zoster by age class. Values with different superscripts significantly differ according to the Kruskal–Wallis test, followed by Dunn’s *post hoc* test (*p* < 0.05).

During the 2009–2023 period, herpes zoster predominantly affected older adults. The occurrence of herpes zoster increased significantly with age, with the highest rates observed in the oldest age group (>65 years) (Figure 1).

This group was significantly more affected than all younger age groups (Dunn’s *post hoc* test, *p* < 0.0001). Patients aged 56–65 years also showed a higher reporting compared to younger adults, though not as pronounced as the >65 group.

**Figure 2:**
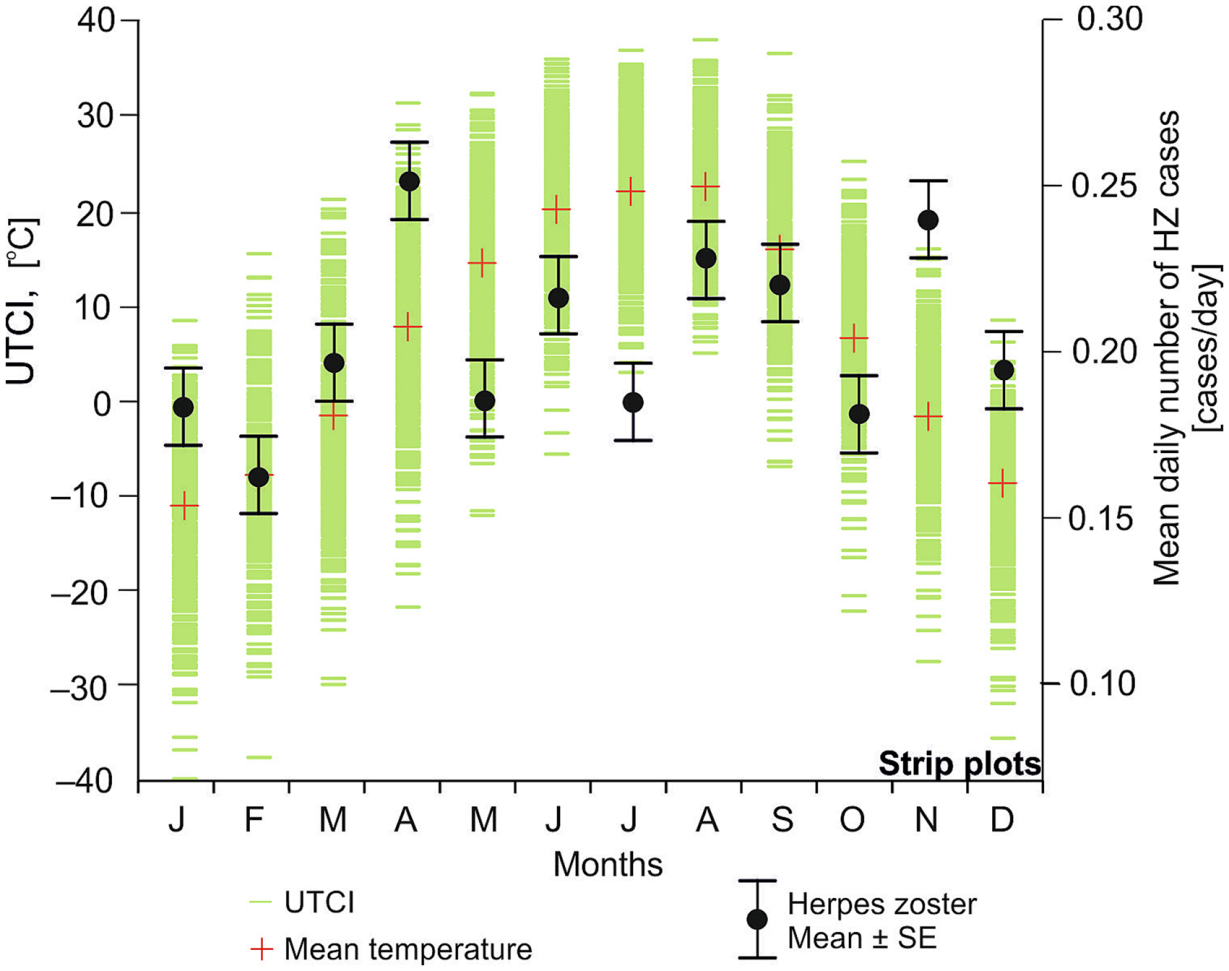
Monthly distribution of the average number of herpes zoster admissions per day (black dots) and whiskers (±SE) on the background of daily UTCI strip plots (green).

No significant seasonal pattern in herpes zoster occurrence was detected. While the average number of cases appeared slightly higher in the summer months, the variation was not statistically significant (Kruskal–Wallis test, *p* > 0.05; Figure 2).

For example, differences in occurrence between February and April or between August and November were not significant, even after adjusting for the different number of days in each month (data not shown). Consistent with the lack of clear seasonality, correlation analysis showed no meaningful association between the number of herpes zoster cases and the meteorological variables examined. None of the Spearman’s correlation coefficients between case counts and factors such as temperature, humidity, precipitation, or wind speed reached statistical significance. Even the correlation with the composite thermal index (UTCI) was essentially zero (*r* = 0.023, *p* > 0.05), indicating a negligible effect of thermal comfort on herpes zoster reporting.

**Figure 3:**
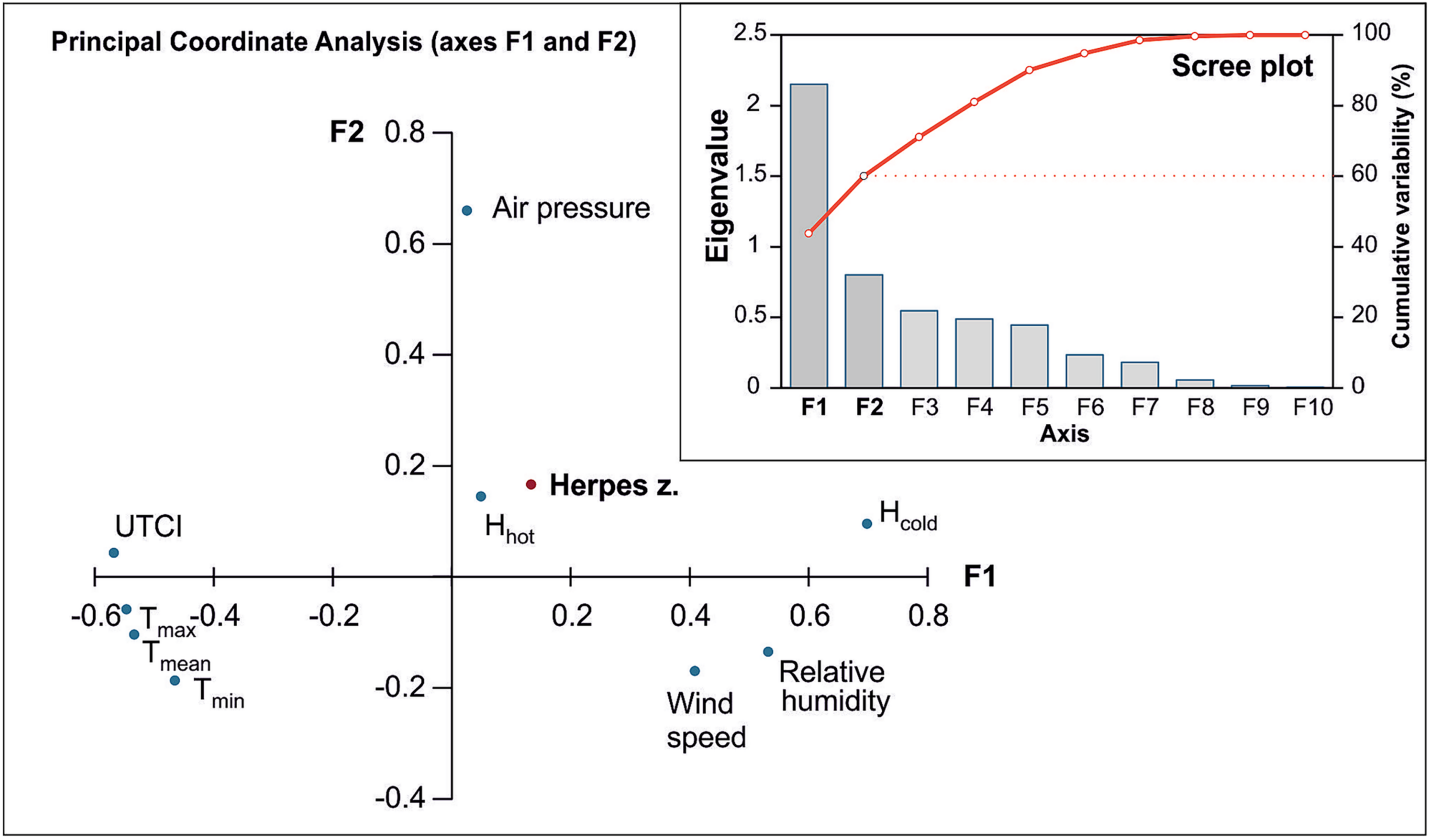
Principal Coordinate Analysis (PCoA) plot showing the relationship between weather variables, the Universal Thermal Climate Index (UTCI), H index, and the occurrence of herpes zoster. The axes (F1 and F2) represent the primary dimensions of variability, with F1 capturing 43.74% and F2 capturing 16.27% of the variance. The scree plot indicates the eigenvalues and cumulative variability explained by each axis, with F1 and F2 together accounting for 60.01% of the variability in the dataset.

The multivariate PCoA provided further insight (Figure 3). In the ordination, the occurrence of herpes zoster (for the total cohort) aligned more closely with certain variables such as the number of days with heat discomfort (H_hot_) and relative humidity (RH), suggesting a positive association with these factors along the first principal coordinate (F1). In contrast, temperature-related variables (mean, maximum, and minimum air temperature, as well as the UTCI) plotted in the opposite direction from herpes zoster occurance on the F1 axis, indicating little to no direct association between simple temperature measures and the occurrence of the disease.

**Figure 4:**
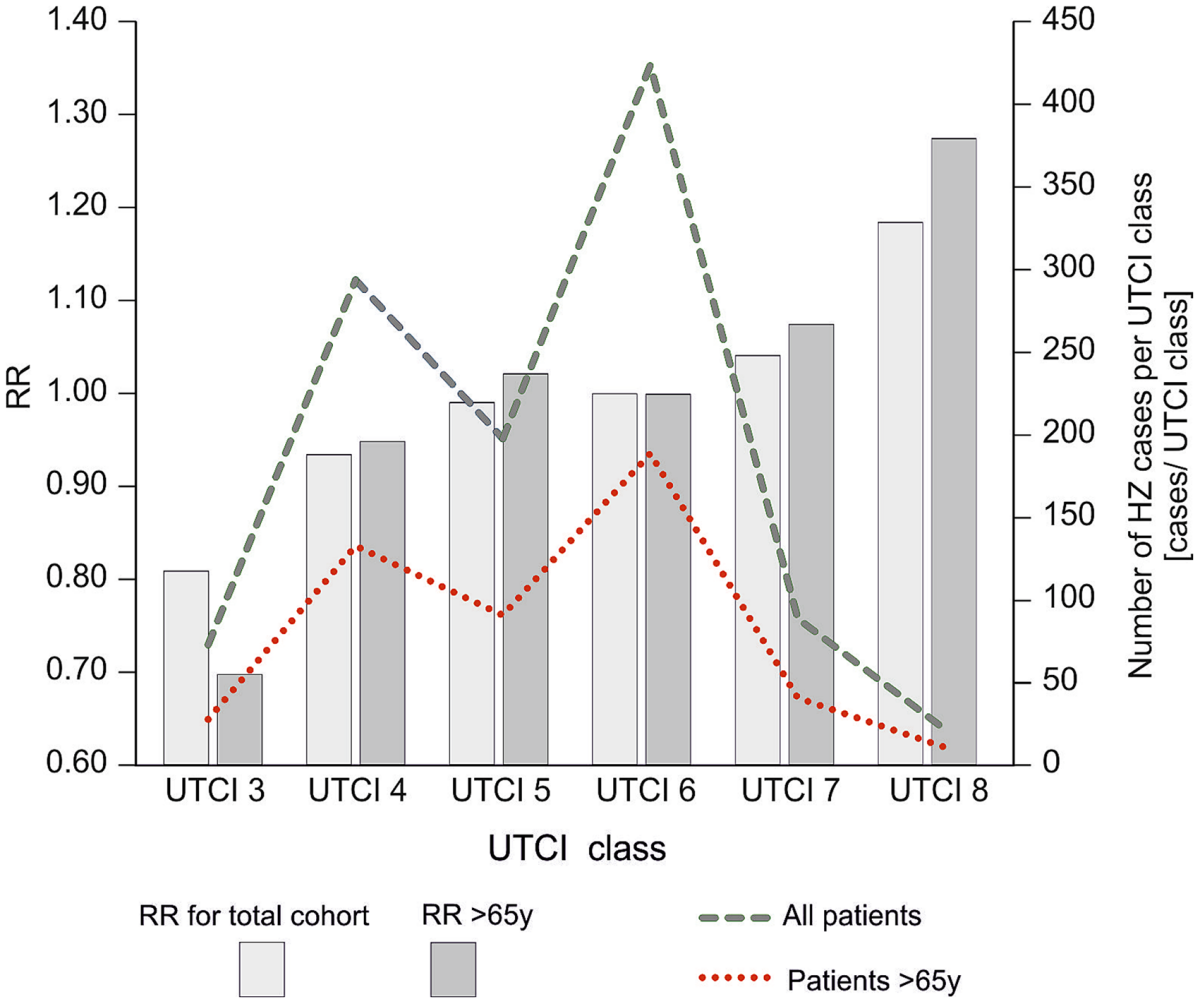
Average daily number of herpes zoster admissions in relation to UTCI class in the general population and in the group of patients aged >65. The lighter bars correspond to the total cohort, while the darker bars represent patients aged >65.

We next examined herpes zoster reporting across categories of thermal conditions as defined by UTCI, comparing the entire study population to the subset of older patients (>65 years) (Figure 4). We observed that extreme heat stress conditions were associated with higher numbers of cases. In both the overall population and in seniors, the average daily number of admissions was lowest under moderate thermal conditions (UTCI class 6) and increased markedly under heat stress conditions (peaking at UTCI classes 7 and 8). The >65 years group had fewer cases per day than the total population at each UTCI category, but showed a similar upward trend across UTCI classes. This indicates that very hot weather conditions correspond to an increase in herpes zoster cases in all adults, although the absolute occurance remains lower in the elderly subgroup.

**Figure 5:**
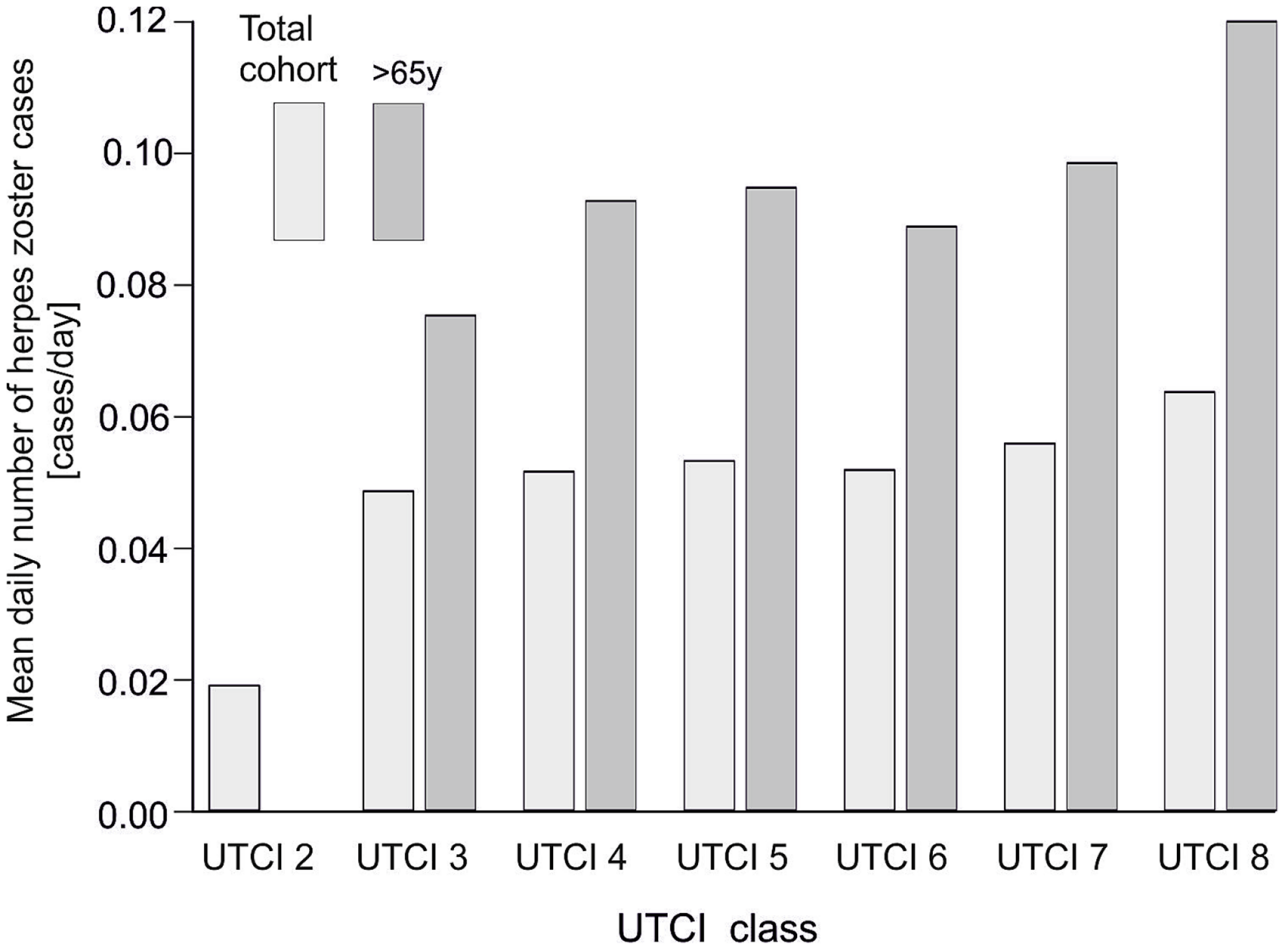
Relationship between herpes zoster admissions and Universal Thermal Climate Index (UTCI) classes. Bars represent the relative risk (RR) of herpes zoster admissions for the total cohort (grey bars) and patients aged >65 years (red dashed line). The dashed green line represents the number of admissions per UTCI class for the total cohort, while the dotted red line represents the number of admissions for seniors aged >65 years. The graph did not show UTCI 2 due to a lack of patients >65 years.

In addition to case counts, we calculated the relative risk of herpes zoster for different UTCI categories (Figure 5; Supplementary Table S2). In the general population, the relative risk of zoster increased steadily with warmer UTCI classes, reaching the highest values under the most extreme heat stress (UTCI class 8). For the >65 age group, a similar trend of rising RR with higher UTCI was observed, though the RR values for seniors were consistently lower than those for the total population at corresponding classes. In both the overall and >65 groups, the relative risk was markedly elevated in heat stress conditions compared to neutral conditions. Concordantly, the number of herpes zoster admissions per UTCI class was highest at the extreme heat category (UTCI class 8) in both populations. Thus, extreme thermal conditions were associated with an increased risk and frequency of herpes zoster, with the effect somewhat attenuated among older individuals. For example, the risk of herpes zoster was approximately 20–25% higher on days classified as strong heat stress (UTCI class 8) compared to thermally neutral days, whereas it was slightly lower under milder cold stress conditions (UTCI class 3), relative to neutral. Notably, these meteorological associations emerged despite the lack of any overt seasonal pattern in reporting, indicating that herpes zoster occurrence is not governed by season per se but can be modulated by specific thermal conditions.

## Discussion

This study examined the relationship between weather conditions and herpes zoster occurrence, with a particular focus on using the composite index UTCI to capture thermal stress. In the overall analysis using straightforward meteorological variables (temperature, humidity, etc.), we did not find any significant association with herpes zoster case numbers, nor any clear seasonal trend. However, when we categorized days by UTCI-defined thermal stress levels, a distinct pattern emerged: periods of heat stress corresponded to higher rates of herpes zoster reactivation, whereas periods of cold stress were associated with lower rates. This contrast suggests that while individual weather parameters alone may not show an effect, the combined effect captured by UTCI can reveal an influence of extreme thermal conditions on herpes zoster occurrence. The multivariate PCoA supports this interpretation, as it showed herpes zoster cases clustering with indices of heat stress in the multidimensional analysis.

Unlike using temperature alone, the UTCI incorporates multiple meteorological factors (temperature, humidity, wind, and solar radiation) to assess human thermal stress, making it a more precise indicator of weather conditions relevant to health outcomes (38). Our findings underscore the value of such a comprehensive index: even when no single weather variable showed a strong effect, the integrated thermal load (as represented by UTCI) was able to distinguish conditions that predispose to viral reactivation. In contrast, the H index did not provide additional insights in our dataset, likely because UTCI integrates multiple meteorological variables and therefore offers a more comprehensive and physiologically relevant measure of thermal stress in this context. The analysis of H index (Supplementary Table S3) nevertheless indicated a significantly increased risk of herpes zoster under very hot conditions, although overall its explanatory value was lower than that of the UTCI. Notably, days meeting the “very hot” H-class criterion were relatively infrequent in our series, which limits the precision of the risk estimate. This finding should therefore be interpreted with caution and corroborated in larger datasets and settings with more frequent extreme heat exposure.

The UTCI has been previously linked to infectious diseases. In Poland, lower UTCI values, indicating stronger cold stress, correlated with higher consultation rates for respiratory infections (32). This supports the hypothesis that thermal stress may influence immune function and susceptibility to viral reactivation, including varicella-zoster virus.

Extreme heat is a well-recognized hazard for public health, and its importance is growing in the context of global climate change (33). For instance, the severe European heatwave of 2003 was associated with around 70,000 excess deaths, predominantly among the elderly (39). Heatwaves not only cause direct heat-related illness and mortality, but they can also aggravate chronic conditions such as cardiovascular and respiratory diseases (40). With climate change, the frequency and intensity of heat extremes are rising; the proportion of heat-related deaths in people over 65 has increased significantly (by an estimated 85% between 2000 and 2021) (41).

Physiological stress from extreme temperatures can influence the immune system and has been implicated in the reactivation of other latent viruses (42). For example, heat stress has been observed to precipitate reactivation of herpes simplex virus (HSV-1) in susceptible individuals (43). A comprehensive review by Kennedy et al. (UK) compared HSV-1 and VZV, demonstrating that both viruses share key mechanisms of latency and reactivation, such as persistence in sensory ganglia and reliance on host immune control. This parallel supports the biological plausibility that similar triggers, such as thermal stress, may also contribute to VZV reactivation. In the case of VZV, our results suggest that elevated ambient temperatures might act as a trigger that disturbs the balance between the virus and the host’s nervous system, allowing the virus to emerge from latency (44). Figures 4, 5 similarly demonstrate that HZ occurrence increases with higher UTCI classes, underscoring a significant effect of thermal stress on VZV reactivation. Marcus et al. (USA) reported that *in vitro*, lowering the incubation temperature from 37 °C to 34 °C markedly increased the reactivation of VZV [36]. Notably, a skin surface temperature around 34 °C can occur under relatively mild heat stress (for example, on a warm day when a person is lightly clothed, corresponding to UTCI classes 7–8, 26–38 °C). Although more extreme heat stress conditions (beyond class 8) were not encountered in our region during the study period, the occurrence of high UTCI values – and the associated physiological heat strain – was clearly linked to increased herpes zoster cases in our analysis (see Figure 3).

Our findings in Figure 2 and Supplementary Table S1 indicate no meaningful relationship between ambient temperature alone and HZ cases, implying temperature by itself is not sufficient to trigger reactivation. The slightly lower number of cases observed in February (relative to some longer months like August or November) is likely explained by February’s shorter duration, and this difference was only on the borderline of statistical significance; thus, it should be interpreted with caution. Overall, an analysis based purely on calendar months or single weather variables could miss important nuances. In contrast, using the UTCI, which combines multiple meteorological factors, provided a clearer picture of risk: it seems that it is the composite effect of temperature together with humidity, wind, and solar radiation (and even behavioral factors like clothing insulation) that contributes to changes in HZ occurrence. UTCI thus appears to be a more effective predictor of HZ reactivation risk than temperature alone.

Our results are in line with several studies that found no strong seasonality in herpes zoster occurrence. Investigations in Australia and South Korea, for instance, reported no significant seasonal pattern in herpes zoster cases, whether examining hospitalizations or emergency department visits (45, 46). On the other hand, Wu et al. (Taiwan, China) noted that there was generally no pronounced seasonality, the number of cases did peak in the hottest month (August), (47). Notably, the climate in the heavily populated region of Taipei (subtropical, Köppen Cfa) features summer temperatures around 28 °C, which corresponds to a moderate heat stress level (approximately UTCI class 7) (31). This observation from Taiwan, China aligns with our finding that conditions of heat stress can increase HZ risk.

The lack of a traditional seasonal pattern in our data supports the notion that it is not the season or the calendar month per se, but rather the actual thermal stress from temperature extremes that drives the risk of disease. Focusing solely on calendar-defined seasons can be misleading, especially as climate change leads to atypical weather (warmer winters, unseasonal heatwaves) that disrupt usual seasonal norms (48). Our findings support the notion that HZ occurrence is not strictly seasonal but is influenced by meteorological conditions. It should be noted, however, that host factors—particularly age—remain fundamental determinants of risk (49). In our study, older adults had higher baseline occurrence of HZ (Figure 1). Even so, when considering our age-specific results alongside the UTCI analysis (Figures 4, 5), we see that thermal stress influences both younger and older adults, highlighting UTCI as a useful predictor of risk across age groups.

These results suggest that it may be worth incorporating complex climatic indices, like the UTCI, into public health planning for HZ prevention. If certain weather conditions indeed heighten the risk of zoster, health authorities could consider timing preventive measures (such as vaccination campaigns) to precede periods of high thermal stress. Vaccination is an effective strategy to reduce the risk of HZ reactivation, and aligning vaccine administration with forecasts of elevated UTCI values might enhance its protective impact (50). To date, vaccination programs are generally organized around fixed schedules or known seasonal patterns for other diseases (for example, annual influenza campaigns) (51, 52), and HZ immunization is primarily recommended based on age or immune status rather than climate considerations (53). This paradigm was evident during the initial rollout of COVID-19 vaccines, where prioritization was based on vulnerability and risk rather than environmental factors (54). Currently, weather conditions are rarely taken into account for vaccinations; HZ vaccines are administered irrespective of temperature or season, focusing mainly on the patient’s age (55). Our findings highlight a potential benefit in considering meteorological risk factors in HZ prevention strategies, especially in the face of changing climate patterns.

In this 2009–2023 study, we observed that days characterized by higher thermal stress (UTCI) coincided with increased herpes zoster risk, indicating that biometeorological conditions may influence VZV reactivation. These results align with evidence from Taiwan and Hefei, China, as well as South Korea, where warmer temperatures were linked to higher herpes zoster reporting or health-care utilization (11, 14, 17). Conversely, data from Shanghai reported winter predominance, highlighting that the direction and magnitude of temperature-related effects may vary by climatic zone, population characteristics, and exposure metrics (15). Notably, our use of the integrative UTCI adds physiological relevance compared with single-parameter indices. Taken together, the literature and our findings support the biological plausibility that heat-related stress can destabilize host–virus equilibrium and facilitate reactivation in susceptible individuals. Given the absence of patient-level confounders (e.g., vaccination, immunosuppression) and the use of regional exposure, these results should be considered hypothesis-generating and call for larger, multicenter studies with finer exposure assessment and richer clinical data.

## Limitations

This study has several limitations. First, because the data were drawn from only two medical facilities, the findings might not be fully generalizable to the entire region or other populations. These two facilities were chosen because they provided complete and standardized electronic medical records for the entire study period, but this restriction, while ensuring high data quality, inevitably limits the generalizability of the findings to broader populations. Second, the meteorological conditions were assigned based on regional weather station data, which may not capture each individual’s exact exposure (microclimates or personal environmental conditions could differ). Third, as a retrospective analysis of medical records, the study relies on the accuracy and consistency of those records; any differences in diagnostic or reporting practices between the two facilities could affect the results. Additionally, we did not have data on certain personal factors such as patients’ immune status, co-morbid conditions, or stress levels, which could influence the likelihood of VZV reactivation. Moreover, vaccination status against varicella or herpes zoster was not available in our dataset and therefore could not be controlled for as a potential confounder. The shingles vaccine became available in Poland in March 2023, but its impact can be disregarded in our retrospective analysis since nationwide access only began on April 1, 2025, when it was added to the list of reimbursed drugs for individuals over 65 years by the National Health Fund (NFZ) (56). Another limitation is the use of regional meteorological data rather than individual-level exposure measures. Such an ecological approach may introduce bias, as personal behaviors (e.g., time spent outdoors, air conditioning use) and microclimatic variations could differ substantially across patients, leading to potential exposure misclassification. Another potential source of classification bias arises from the use of ICD-10 codes, which do not allow full distinction between recurrent or prolonged cases. To reduce this risk, repeated consultations occurring within 30 days were excluded from the analysis, but some residual misclassification cannot be entirely ruled out. UCTI may overestimate cold stress in populations that adapt through protective clothing or heating, while at the same time underestimating true exposure variability when based on single daily measurements. Future research should consider incorporating these clinical variables and possibly a broader range of locations to provide a more comprehensive understanding of how weather and other factors interact to affect herpes zoster risk.

## Conclusion

Our findings indicate that thermal stress, particularly heat stress captured by the UTCI, may play a role in shaping herpes zoster occurrence, even in the absence of clear seasonality. This work adds to the emerging evidence linking climatic conditions with infectious diseases, offering a new perspective on herpes zoster epidemiology. While the study is exploratory and constrained by its retrospective design, limited geographic scope, and absence of certain individual-level variables, it highlights important avenues for future research. Expanding analyses to larger, multicenter cohorts and integrating data on vaccination, immune function, and behavioral exposures will be essential to confirm and refine these associations. Ultimately, a deeper understanding of the interplay between climate variability and herpes zoster risk may inform both preventive strategies and public health planning in the era of global climate change.

## Data Availability

The raw data supporting the conclusions of this article will be made available by the authors, without undue reservation.
