# Supplementary material for: Influence of meteorological conditions on herpes zoster occurrence: a retrospective cohort study
Source: Front Med (Lausanne). 2025 Oct 10;12:1643828. doi: 10.3389/fmed.2025.1643828 (PMC12549569; doi:10.3389/fmed.2025.1643828)
Supplement: Supplementary file 1 [file Table_1.DOCX]

Table S1 **Relative risk (RR) of herpes zoster according to Universal Thermal Climate Index (UTCI) classes for the study cohort (upper table) and for patients group aged >65 years (lower table).**

The tables present relative risk values with 95% confidence intervals (–95% CI, +95% CI), together with Z-scores and p-values obtained from pairwise comparisons. RR values >1 indicate a higher risk of herpes zoster in the given UTCI class compared with the reference category, whereas RR <1 indicates a lower risk. UTCI class 6 (9–26 °C, mild thermal stress) was used as the reference category.

| **All patients included in the study** | | | | | |
| --- | --- | --- | --- | --- | --- |
| **UTCI class** | **RR** | –95% CI | +95% CI | Z-score | p |
| **2** | **0.28** | 0.220 | 0.366 | 9.658 | **0.000** |
| **3** | **0.81** | 0.698 | 0.946 | 2.683 | **0.004** |
| **4** | **0.93** | 0.811 | 1.077 | 0.932 | 0.176 |
| **5** | **1.00** | 0.868 | 1.142 | 0.069 | 0.473 |
| **6** | **1.00** | Reference class |  |  |  |
| **7** | **1.04** | 0.912 | 1.193 | 0.619 | 0.268 |
| **8** | **1.14** | 1.002 | 1.295 | 1.993 | **0.023** |
|  |  |  |  |  |  |
|  |  |  |  |  |  |
| **Group >65 years** | | | | | |
| **UTCI class** | **RR** | –95% CI | +95% CI | Z-score | p |
| **2** |  |  |  |  |  |
| **3** | **0.70** | 0.559 | 0.874 | 3.134 | **0.001** |
| **4** | **0.95** | 0.785 | 1.140 | 0.581 | 0.281 |
| **5** | **1.03** | 0.861 | 1.227 | 0.308 | 0.379 |
| **6** | **1.00** | Reference class |  |  |  |
| **7** | **1.08** | 0.906 | 1.277 | 0.828 | 0.204 |
| **8** | **1.28** | 1.096 | 1.489 | 3.129 | **0.001** |
