# Supplementary material for: Influence of meteorological conditions on herpes zoster occurrence: a retrospective cohort study
Source: Front Med (Lausanne). 2025 Oct 10;12:1643828. doi: 10.3389/fmed.2025.1643828 (PMC12549569; doi:10.3389/fmed.2025.1643828)
Supplement: Supplementary file 2 [file Table_2.DOCX]

Table S2 **Relative risk (RR) of herpes zoster according to Cooling Power Index (H) classes for the study cohort (upper table) and for patients group aged >65 years (lower table).**

The tables present relative risk values with 95% confidence intervals (–95% CI, +95% CI), together with Z-scores and p-values obtained from pairwise comparisons. RR values >1 indicate a higher risk of herpes zoster in the given H class compared with the reference category, whereas RR <1 indicates a lower risk. H class neutral (210-1260 W/m^2^) was used as the reference category.

| **All patients included in the study** | | | | | |
| --- | --- | --- | --- | --- | --- |
| **H class** | **RR** | –95% CI | +95% CI | Z-score | p |
| H _very hot_  (<210 W/m^2^) | 1.44 | 1.245 | 1.658 | 4.957 | **<0.0001** |
| H _neutral_  (210-1260 W/m^2^) | 1.00 | Reference class |  |  |  |
| H_cold_  (>1260 W/m^2^) | 0.93 | 0.785 | 1.095 | 0.891 | 0.187 |
|  |  |  |  |  |  |
