# Supplementary material for: Influence of meteorological conditions on herpes zoster occurrence: a retrospective cohort study
Source: Front Med (Lausanne). 2025 Oct 10;12:1643828. doi: 10.3389/fmed.2025.1643828 (PMC12549569; doi:10.3389/fmed.2025.1643828)
Supplement: Supplementary file 3 [file Table_3.DOCX]

Table S3 – Spearman correlation matrix (p<0.05) between herpes zoster admissions (N=5478) and meteorological parameters, the UTCI and H in the period of observation (2009-2023).

| Variable | Herpes zoster | UTCI | Tmean | Precipi-  tation | Wind speed | Pressure | Relative humidity | T_max_ | T_min_ | H_hot_ | H_cold_ |
| --- | --- | --- | --- | --- | --- | --- | --- | --- | --- | --- | --- |
| Herpes zoster | **1** | 0.023 | 0.020 | 0.005 | -0.013 | -0.006 | -0.010 | 0.020 | 0.023 | 0.015 | -0.016 |
| UTCI |  | **1** | **0.917** | **-0.127** | **-0.402** | **0.115** | **-0.515** | **0.939** | **0.815** | **0.121** | **-0.772** |
| Air temperature, T_mean_ |  |  | **1** | 0.012 | **-0.166** | -0.026 | **-0.455** | **0.984** | **0.952** | **0.120** | **-0.669** |
| Precipitation |  |  |  | **1** | **0.211** | **-0.432** | **0.366** | **-0.030** | **0.101** | 0.007 | **0.093** |
| Wind speed |  |  |  |  | **1** | **-0.265** | **0.028** | **-0.197** | **-0.085** | **-0.035** | **0.457** |
| Air pressure |  |  |  |  |  | **1** | **-0.212** | 0.016 | **-0.112** | 0.003 | **-0.110** |
| Relative humidity |  |  |  |  |  |  | **1** | **-0.531** | **-0.283** | **-0.085** | **0.280** |
| T_max_ |  |  |  |  |  |  |  | **1** | **0.903** | **0.121** | **-0.675** |
| T_min_ |  |  |  |  |  |  |  |  | **1** | **0.117** | **-0.610** |
| H_hot_ |  |  |  |  |  |  |  |  |  | **1** | **-0.050** |
| H_cold_ |  |  |  |  |  |  |  |  |  |  | **1** |

UTCI – Universal Thermal Climate Index,
Tavr – average air temperature,
Tmax – maximum air temperature,
Tmin – minimum air temperature,
H_hot_ – number of days with heat discomfort; Cooling power index <210 W/m^2^
H_cold_ – number of days with cold discomfort; Cooling power index <1260 W/m^2^
